# Absolute and relative quantification of RNA modifications *via* biosynthetic isotopomers

**DOI:** 10.1093/nar/gku733

**Published:** 2014-08-16

**Authors:** Stefanie Kellner, Antonia Ochel, Kathrin Thüring, Felix Spenkuch, Jennifer Neumann, Sunny Sharma, Karl-Dieter Entian, Dirk Schneider, Mark Helm

**Affiliations:** 1 Institute of Pharmacy and Biochemistry, Johannes Gutenberg University Mainz, 55099 Mainz, Germany; 2 Institute for Molecular Biosciences, Johann-Wolfgang Goethe University, 60438 Frankfurt am Main, Germany

## Abstract

In the resurging field of RNA modifications, quantification is a bottleneck blocking many exciting avenues. With currently over 150 known nucleoside alterations, detection and quantification methods must encompass multiple modifications for a comprehensive profile. LC–MS/MS approaches offer a perspective for comprehensive parallel quantification of all the various modifications found in total RNA of a given organism. By feeding ^13^C-glucose as sole carbon source, we have generated a stable isotope-labeled internal standard (SIL-IS) for bacterial RNA, which facilitates relative comparison of all modifications. While conventional SIL-IS approaches require the chemical synthesis of single modifications in weighable quantities, this SIL-IS consists of a nucleoside mixture covering all detectable RNA modifications of *Escherichia coli*, yet in small and initially unknown quantities. For absolute in addition to relative quantification, those quantities were determined by a combination of external calibration and sample spiking of the biosynthetic SIL-IS. For each nucleoside, we thus obtained a very robust relative response factor, which permits direct conversion of the MS signal to absolute amounts of substance. The application of the validated SIL-IS allowed highly precise quantification with standard deviations <2% during a 12-week period, and a linear dynamic range that was extended by two orders of magnitude.

## INTRODUCTION

Accurate quantification of modified nucleosides at high sensitivity has become a pressing problem, as increasing evidence suggests widespread, if not ubiquitous occurrence of nucleoside modifications in RNA. While earlier studies focused on abundant species such as rRNA and tRNA (1,2), it has recently become clear that modifications in mRNA and low abundant ncRNA hold a high potential for regulation of gene expression (3–7). In addition, RNA modifications appear no longer as mere concrete-cast equipment to chemically enhance the performance of components of the translation system. Instead, a notion of dynamics has entered the field, as the modification patterns of tRNA populations were found to vary as a function of various types of stress (8). Modifications in mRNA can actually be removed as well as added, a concept assimilated from the related field of DNA modifications, along with a connection to epigenetic phenomena (9,10).

While early studies occasionally noticed incomplete modification at a given position (11,12), occurrence of modified nucleosides was essentially treated in a binary yes/no perspective. The exciting recent developments call for methods to determine the modification yield in a given RNA with more precision. Assuming a single species, e.g. a tRNA isoacceptor or an rRNA (13), can be isolated in sufficient purity, established protocols for the determination of nucleoside modifications make quantification of modifications in relative, or even absolute terms appear straightforward at first glance. The most advanced approach that conserves sequence information relies on liquid chromatography–mass spectrometry (LC–MS) analysis of fragments obtained from complete hydrolysis by RNase T1 (14–16), but the sensitivity of this technique is currently lagging behind nucleoside quantification by several orders of magnitude. Other standard protocols for quantification include digestion of the RNA sample to monomers, which are measured after separation by chromatography. Most importantly for the problem at hand, i.e. for accurate quantification, the signal obtained for each individual modification (or nucleotide) must be correlated to the corresponding amount of substance. In the optimal dynamic range, this correlation is linear and therefore affected by simple multiplication with a response factor (RF). The robustness of response factors depends on a variety of aspects, and this is, in essence, what this entire paper is concerned with.

For example, early methods employed *in vivo* labeling of RNA, e.g. by cell culture in the presence of ^32^P. In experiments including complete digestion to mononucleotides, the latter can be separated by two-dimensional thin layer chromatography, and quantified after exposure to phosphoimager screens (12). Provided the label is evenly spread throughout the nucleoside-triphosphate (NTP) pool of the cell, this method yields an identical response factor for every nucleotide (modification), although absolute amounts of substance cannot be determined.

**Figure 1.**
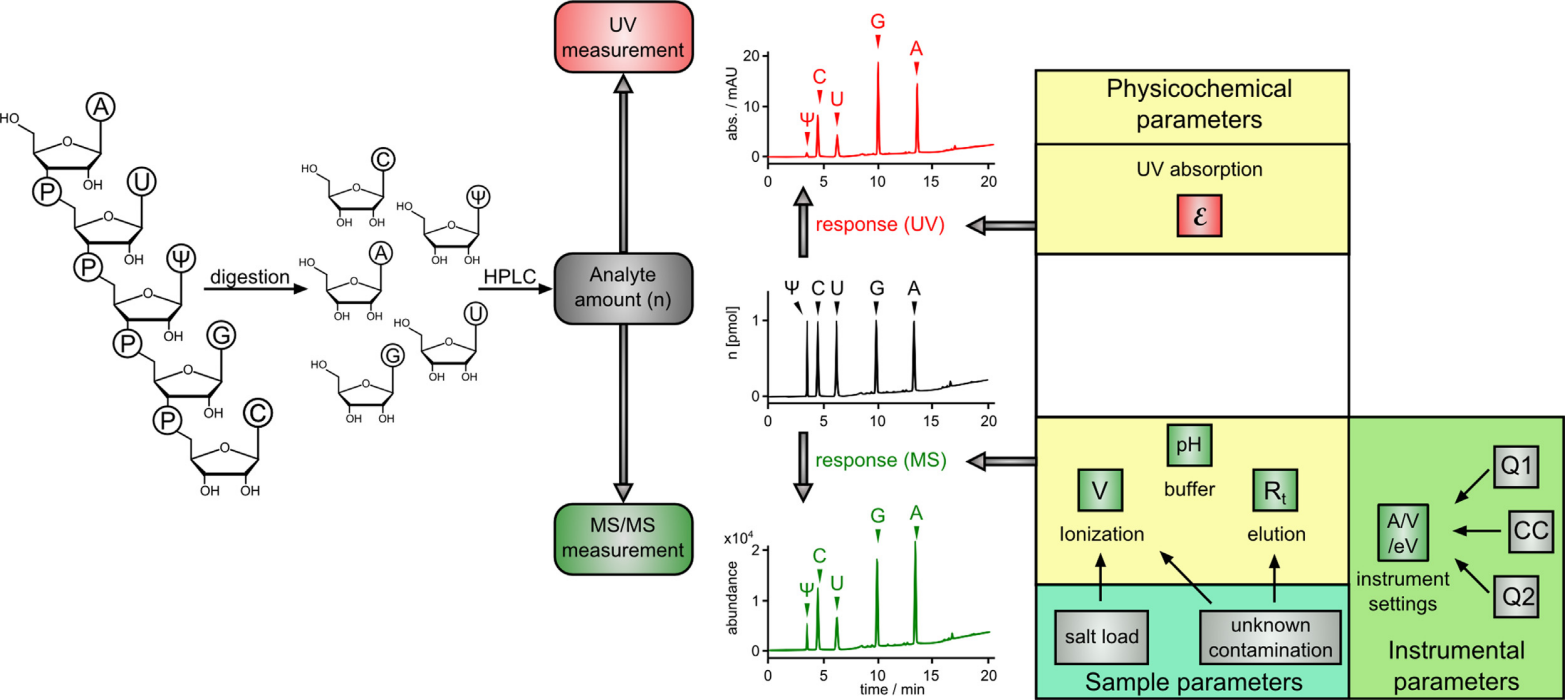
Correlation of absolute analyte amount and signal intensity using UV and MS/MS measurements. As an example, an oligomer with equimolar amounts of each depicted nucleoside is digested and separated by column chromatography. The chromatograms above (UV) and below (MS/MS) represent the effect of the response factors on the signal intensities. The UV signal is essentially dependent on only the absorption coefficient *ϵ*, which is mildly dependent on solvent composition and pH. The MS/MS signal is subject to significant and varying influence by several physicochemical properties, sample parameters and instrumental parameters that cannot be assessed in one generally applicable parameter (legend: V, volt; R_t_, retention time; A, ampere; eV, electron volt; Q1(2), quadrupol1(2) and CC/collision cell).

Quantification methods that eschew the use of radioactivity may be based on UV-absorption for quantification. Digestion to nucleosides is followed by separation on an RP-18 HPLC column, and detection relies on a UV detector, typically at 254 nm. As depicted in Figure 1, quantification is based on Lambert Beer's law, which invokes the extinction coefficient *ϵ* to account for differential detection efficiency of various nucleosides. Extinction coefficients are only mildly dependent on solvent and pH, and therefore variations of extinction-based response factors in a freshly calibrated system are minimal. These are prototypes of robust response factors, and are typically obtained by external calibration, i.e. from a dilution series of known amounts of substance for each given nucleoside. However, there is an intrinsic lower limit to the detection of nucleosides somewhere in the single-digit picomole range, which makes it unsuitable for most of the current pressing problems in the field. Currently, most sensitive and accurate quantification methods rely on mass spectrometry. LC–MS/MS techniques using triple quadrupole-based detection allow limits of quantification in the low femtomole range (17,18). However, compared to UV-based quantification, response factors in MS are considerably less robust. The left side of Figure 1 outlines the difficulties for quantification with MS/MS systems for which we suggest a solution in this paper. Parameters that affect the response factors fall into three categories, namely (i) instrumental parameters, (ii) sample parameters and (iii) physicochemical parameters of the analyte/solvent system. A point in case is the recent report by the Limbach group, in which the reproducibility of modified nucleoside quantification by LC–UV/MS in four replicative measurements has been analyzed. For MS detection, the average percentage of relative standard deviation (RSD) in peak areas measured for the ions was 5.9%. RSD variability in MS peak areas in this study ranged from 1.0 to 12.4% (16).

Current analytical tasks in the field include, e.g. comparative quantification of modifications among several samples (8,19,20), absolute quantification of modifications in a mixture of RNA species, and quantification of the relative occupancy of modifications in a pure RNA species. In the latter two, the modification yield is typically given in moles modification per mole parent nucleoside (21) or moles tRNA (22), which necessitates to relate the sample signal to an absolute amount. This, in turn, requires calibration with standard solutions obtained from weighable amounts of material, and is typically performed as external calibration (23) or spike-in measurements (24). Since we have repeatedly encountered inconsistent results with these techniques we turned to the use of an internal standard (IS). Although single nucleoside species have been used as general IS for all modifications in a given sample (19,25), we maintain that accurate response factors can only derive from isotopomers for each specific modification (26), obtained by stable isotope labeling (SIL) (27–29). The Carell laboratory has recently published impressive quantification results (30,31) based on a series of isotope-labeled internal standards obtained by means of synthetic organic chemistry. By this approach, determination of absolute substance amounts in samples is possible because the isotopomers are available in quantities sufficient for accurate weighing. However, each compound represents a significant amount of synthetic work, and availability is limited by synthetic routes to the target compounds. For nucleoside modifications naturally occurring in RNA, this is a serious problem, since they occur in great chemical diversity. For example, several dozens of different modifications are present in *E. coli* (20,32), and synthesis of an SIL-IS for every single one of these constitutes an obvious limitation for most of the laboratories.

As a fast and experimentally straightforward solution to this problem, we present a strategy for the generation, validation and application of a comprehensive SIL-IS, which encompasses all relevant modifications occurring in a given organism, here in *Escherichia coli*. The labeled nucleoside standards were isolated from cultures raised in the medium containing ^13^C glucose as the only carbon source. A digestion to nucleosides generates a mixture of labeled nucleosides which can be used for the relative comparison of two samples without further calibration effort. To allow for absolute quantification in addition, the initially unknown amounts of the various modifications in the SIL-IS mixture were determined and validated *via* a combination of external calibration, internal standard and spike-in methods. This accounts for variations of instrumental parameters, physicochemical parameters, and in particular of sample parameters. Consequently, the method allows, in addition to comparison of the relative modification content of related samples, an absolute quantification, limited to those nucleosides available in weighable quantities. The method also extends the linear dynamic range for quantification by two orders of magnitude by equalizing ion suppression effects due to saturation.

## MATERIALS AND METHODS

### Preparation of internal standard

#### Growth of *E. coli* in ^13^C glucose medium and isolation of total RNA

*Escherichia coli* (strain MC4100) was grown in M9 medium (2 mM MgSO_4_, 0.1 mM CaCl_2_, 0.4% glucose, 6.8 g/l Na_2_HPO_4_, 3 g/l KH_2_PO_4_, 0.5 g/l NaCl and 1 g/l NH_4_Cl). The medium contained ^13^C-glucose (all carbons exchanged for ^13^C, from Sigma-Aldrich, Munich, Germany) as the only carbon source for complete ^13^C labeling of RNAs. The growth of the cultures was monitored, and cells were harvested at an OD_600_ of 1.8 by centrifugation. RNA was isolated using TRI-reagent and the included RNA isolation manual (Sigma-Aldrich, Munich, Germany). All other chemicals needed for isolation were purchased from Sigma-Aldrich, Munich, Germany.

For the preparation of SIL-IS, the concentration of precipitated RNA was measured *via* Nanodrop-ND-2000 (Peqlab, Erlangen, Germany). The RNA was then digested into nucleosides using the following protocol.

Five hundred micrograms RNA (final concentration 1 μg/μl) was incubated at 37°C for 2 h in the presence of 20 U nuclease P1 (Roche Diagnostics, Mannheim, Germany), 1/10 volume of 10× nuclease P1 buffer (0.2 M NH_4_OAc pH 5.0, ZnCl_2_ 0.2 mM) and 5 U snake venom phosphodiesterase (Worthington, Lakewood, USA). After addition of 1/10 volume of 10× fast alkaline phosphatase buffer (Fermentas, St Leon-Roth, Germany) and 15 U fast alkaline phosphatase (Fermentas, St Leon-Roth, Germany), the digest was incubated for 1 h at 37°C and adjusted by adding pure water to a final concentration of 0.1 μg/μl.

Following our standard operating procedure 1/10 volume of the 10× concentrated SIL-IS is added to the samples. In 10 μl, which is the ideal injection volume, 100 ng *E. coli*^13^C RNA digest is being injected.

### Procedure for LC–MS/MS measurements

The samples were analyzed on an Agilent 1260 series equipped with a diode array detector (DAD) and Triple Quadrupole mass spectrometer Agilent 6460. A Synergy Fusion RP column (4 μm particle size, 80 Å pore size, 250 mm length, 2 mm inner diameter) from Phenomenex (Aschaffenburg, Germany) was used at 35°C. The solvents consisted of 5 mM ammonium acetate buffer adjusted to pH 5.3 using acetic acid (solvent A) and pure acetonitrile (solvent B). The elution started with 100% solvent A followed by a linear gradient to 8% solvent B at 10 min and 40% solvent B after 20 min. Initial conditions were regenerated by rinsing with 100% solvent A for 10 min. The flow rate was 0.35 ml/min. The effluent from the column was first measured photometrical at 254 nm by the DAD followed by the mass spectrometer equipped with an electrospray (ESI) ion source (Agilent Jet Stream). ESI parameters were as follows: gas temperature 300°C, gas flow 5 l/min, nebulizer pressure 35 psi, sheath gas temperature 350°C, sheath gas flow 12 l/min, capillary voltage 3500 V. The MS was operated in the positive ion mode using Agilent MassHunter software in the dynamic MRM (multiple reaction monitoring) mode. Nucleoside modifications were identified by a combination of retention time and fragmentation pattern, which included the loss of a ribose or methylated ribose in most cases, except for pseudouridine which was identified by its particular fragmentation in addition to its retention time. The monitored mass transitions, instrument settings and retention time windows can be seen in Supplementary Table S1.

### Preparation of calibration solutions with and without SIL-IS

Synthetic modified nucleosides for preparation of calibration solutions were purchased from: Sigma-Aldrich, Munich, Germany: cytidine (C), uridine (U), guanosine (G), adenosine (A), 5-methylcytidine (m^5^C), 2′-O-methylcytidine (Cm), 4-thiouridine (s^4^U), 5-methyl-2-thiouridine (m^5^s^2^U), 7-methylguanosine (m^7^G), 2′-O-methyladenosine (Am), 1-methyladenosine (m^1^A). 6-Dimethyladenosine (m^6^_2_A) and inosine (I); Berry&Associates, Dexter, MI, USA: 5-methyluridine (m^5^U), pseudouridine (Ψ), 2′-O-methyluridine (Um) and 2′-O-methylguanosine (Gm). 6-Methyladenosine (m^6^A) was a gift from Glenn Björk. Each nucleoside powder was weighed into a clean tube (5–10 mg per nucleoside) and dissolved in pure water to reach a final concentration of 10 mM. The nucleosides were then mixed in a 100 μM solution (major nucleosides final concentration in the mix: 1000 μM). This calibration mix stock solution was then used to prepare 15 calibration solutions in the range of 1 pM (1 amol/μl) to 10 μM (100 pmol/μl). Two sets of these calibration solutions were prepared, either containing 10% of the prepared ^13^C SIL-IS or not. The solutions were then analyzed as described and the measurements repeated after 1, 2, 4, 10 and 12 weeks to assess the methods reproducibility. In between measurements, the calibration solutions were stored at −20°C.

### TruB conversion of *S. cerevisiae* tRNA^Phe^ and quantification of turnover efficiency

tRNA^Phe^ from yeast was produced by *in vitro* transcription (33) and incubated with recombinant TruB (34) in MST 1× buffer (20 mM Tris pH 7.5, 60 mM KCl, 0.02% Tween-20 at 80°C for 70 min). For release of the RNA from the enzyme, the reaction mixture was boiled for 10 min at 95°C and subsequently dissolved in 20 mM NH_4_OAc pH 5.3. The samples were incubated for 2 h at 70°C in the presence of 0.0003 U nuclease P1 (Roche Diagnostics, Mannheim, Germany) per 10 pmol RNA, which leads to a complete degradation to mononucleotides. Snake venom phosphodiesterase (Worthington, Lakewood, USA) was then added to a concentration of 0.06 U per 100 μg RNA, and the mixture was incubated at 37°C for another 1 h. Finally, to convert the resulting mixture of mononucleotides to free nucleosides, 1/10 volume of 10× FastAP buffer (Fermentas, St Leon-Roth, Germany) was added, followed by 3/20 volume of H_2_O, and 1 U of FastAP Thermosensitive Alkaline Phosphatase (FastAP stock at 1 U/μl; from Fermentas, St Leon-Roth, Germany). The mixture was incubated for 1 h at 37°C and divided into two aliquots. One aliquot was spiked with 100 fmol pseudouridine, whereas the other was spiked with pure water, prior to adding 10 vol% of SIL-IS and subjecting the samples to LC-MS/MS analysis.

### Preparation of ribosomal RNA fragments

18S ribosomal RNA from *Saccharomyces cerevisiae* was isolated from strains BY4741 (WT1) and strain XYZ (WT2) from EUROSCARF (http://web.uni-frankfurt.de/fb15/mikro/euroscarf/). Isolation of polysomes and rRNA fragment preparation by mung bean nuclease protection method and rRNA digestion were performed as described previously (35). Ten vol% SIL-IS were added to both samples and analyzed by LC–MS/MS. For relative quantification, each nucleoside MS signal was first divided by the signal of its corresponding isotopomer and subsequently by the UV-signal sum of the four canonical nucleosides to account for differences in the injected sample amounts. The ratio of WT1 to WT2 displays the fold change in the modification level. Absolute quantification was achieved by using the established relative response factors as described in the ‘Results’ section.

## RESULTS

### Quantification with external standard calibration

To establish a relation between the MS signal from an LC-MS run and an absolute amount of substance, external calibration of 10 modified nucleosides, available in weighable quantities, was performed. Solutions of defined concentrations, and dilutions of each of these nucleoside solutions were prepared in the range of 1 amol/μl–10 pmol/μl, as described in the ‘Materials and Methods’ section. The solutions were analyzed with a dynamic MRM (dMRM) method (mass transitions and retention time windows are listed in Supplementary Table S1). In contrast to our rather generic neutral loss scan (NLS) method previously reported (20), individual modifications were now specifically addressed by their retention time and mass transitions. Table 1 shows the limit of detection (LOD) and limit of quantification (LOQ) using the dMRM method, and, in comparison, the LOQs of our recently published NLS method (20). As expected, the LODs are significantly lower when individual nucleosides are monitored under optimized fragmentation conditions (dMRM), compared to NLS where all possible molecules are detected by scanning for a ribose loss. Among all 10 analyzed nucleosides, pseudouridine has the highest LOD and LOQ with 10 and 20 fmol, respectively. All other nucleosides can be detected in the single-digit femtomole range or slightly better. The best detectable nucleoside is 7-methylguanosine with an LOD of 10 amol and an LOQ of only 50 amol. These values for LODs and LOQs clearly demonstrate that the analytical method is, in principle, suitable for highly sensitive detection of modified nucleosides in low abundant RNA species. Several additional modifications from total tRNA digests (*E. coli* or *S. cerevisiae*), namely dihydrouridine (D), 3-methylcytidine (m^3^C), 2-methyladenosine (m^2^A), 1-methylguanosine (m^1^G) and 2-methylguanosine (m^2^G), could also be detected by the dMRM method. However, in the absence of weighable amounts of pure substance, no external calibration is possible to ultimately relate the corresponding LC–MS signal to an absolute amount. Thus, by external calibration, we can compare the content of these species such as D, m^3^C, m^2^A, m^1^G and m^2^G among several samples, but their absolute amounts cannot be determined and sample parameters (Figure 1) cannot be accounted for. The method developed thus far, can also not account for instrumental parameter variations occurring over time. For example, we noticed that, when measuring series of large sample numbers (typically >20), the external calibration tended to become unstable especially in the lower concentration range and had to be repeated between samples (data not shown).

**Table 1.** Limit of detection (LOD) and limit of quantification (LOQ) for 10 modified nucleosides by a dMRM method

		Current method dMRM		NLS method (20)
Name		LOD (fmol)	LOQ (fmol)	LOD (fmol)
Pseudouridine	Ψ	10	20	100
5-Methyluridine	m^5^U	10	20	10
Inosine	I	2	5	10
5-Methylcytidine	m^5^C	1	2	10
6-Dimethyladenosine	m^6^_2_A	1	2	10
2′-O-Methylguanosine	Gm	0.5	1	10
2-Methylguanosine	m^2^G	0.5	1	10
2′-O-Methylcytidine	Cm	0.2	0.5	10
1-methyladenosine	m^1^A	0.1	0.5	100
2′-O-Methyladenosine	Am	0.1	0.5	10
7-Methylguanosine	m^7^G	0.01	0.05	100

LOD values previously obtained (20) in a neutral loss scan are given for comparison.

Furthermore, we noticed that results obtained from biological samples strongly depended on the RNA isolation protocol, and measurements had to be confirmed by spiking known amounts of substance into the sample run. However, a significant drawback of spiking is that a one-time spike in experiment suffers from inherent imprecision, while a multi-point calibration consumes multiple aliquots of sample. Thus, inspired by recent developments in the Carell laboratory (27,30), we turned our attention developing SIL-IS methods.

### Development of an internal standard (SIL-IS)

**Figure 2.**
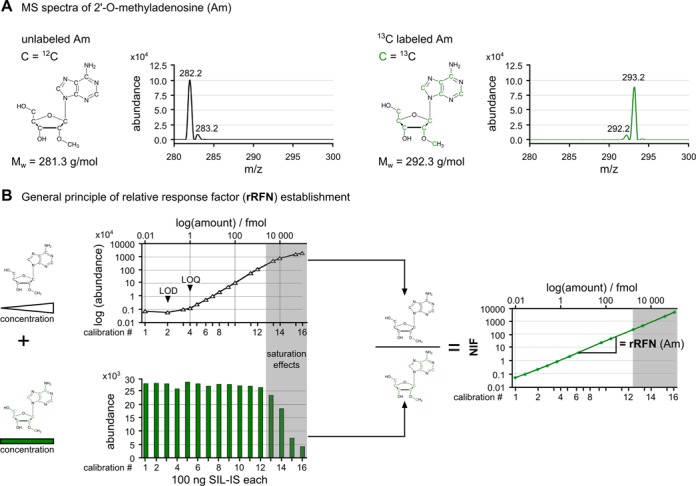
Determination of response factors by usage of commercial Am and ^13^C Am from SIL-IS. (**A**) MS spectrum of unlabeled 2′-O-methyladenosine (Am) (left) and ^13^C-labeled Am (right). (**B**) Upper left: calibration measurements of commercially available, unlabeled Am. At high substance amounts, a flattening of the calibration curve due to saturation effects is highlighted in gray. Below, the signal intensity for constant amounts of ^13^C-labeled Am in the presence of increasing unlabeled Am amounts is shown. Here, the drop in signal intensity due to saturation can also be observed in the gray area. Right: By division of corresponding MS signals of unlabeled and ^13^C labeled Am, the NIF is received and a dynamic calibration curve can be plotted. The slope of the linear fit represents the relative response factor for Am = rRFN (Am).

To obtain a maximum number of isotope-labeled modified nucleosides within a minimum amount of time, we adopted methods from the nuclear magnetic resonance (NMR) field (36–38), where quantitative labeling of nucleosides is commonly performed by feeding bacterial cultures with metabolites containing the isotope of choice. Stable ^13^C isotope-labeled internal standards (SIL-IS) were obtained from *E. coli* cultures grown with ^13^C-labeled glucose as the sole carbon source. The RNA was extracted and digested to the nucleoside level as outlined in detail in the ‘Materials and Methods’ section, to yield a mixture of ^13^C-labeled nucleosides. Mass spectrometric scanning of this mixture by LC–MS/MS revealed an increase in the *m*/*z* ratio of each nucleoside that corresponds precisely to the numbers of carbon atoms in the respective structure. The example of Am is detailed in Figure 2A, where the mass spectrum of the unlabeled nucleoside shows an [M+H]^+^ peak of 282.2, and a typical isotope peak at 283.2, reflecting the 1.1% natural occurrence of ^13^C. In the mass spectrum of the ^13^C-labeled compound, the [M+H]^+^ signal is increased by 11 units, corresponding exactly to the number of carbons in the structure. A small isotope peak at 292.2 reflects a minute fraction of ∼1% ^12^C atoms still present, corresponding to a labeling efficiency of 99%.

The digested ^13^C RNA nucleoside mixture was then used as an SIL-IS for quantification of corresponding, unlabeled nucleosides. This includes the precise addition of SIL-IS to samples in a well-defined ratio, e.g. 10% in our case. The correspondingly adapted dMRM method includes simultaneous measurements of the coeluting sample ^12^C-isotopomers and the SIL-IS ^13^C-isotopomers for each nucleoside modification, whose ratios will be henceforth called nucleoside-isotope factor (NIF**).** Since labeled and unlabeled nucleosides have identical physicochemical properties, the influence of instrumental, physicochemical and sample parameters (Figure 1), including in particular contaminations that may suppress ionization, affect detection of both species equally strong, i.e. the NIF is unaffected. Therefore, the relative nucleoside content of two samples is easily accessible *via* normalization to the ^13^C-isotopomer signal.

More importantly, this method allows precise backtracking of absolute amounts of each modification in the sample, provided the amount of the modification in the SIL-IS mixture is known, or the NIF can otherwise be related to absolute amounts. Therefore, a thorough characterization of the NIF for each modified nucleoside was the logical next step.

### Absolute quantification by combination of external and internal standard calibration

To relate the modification content of the SIL-IS preparation to absolute amounts of substance, a correlation to weighed samples needed to be established. We exploited that ^13^C-isotopomers can be measured side-by-side with the ^12^C-isotopomers in calibration solutions by preparing serial dilutions of an all-in-one mixture of the weighed nucleosides, which were then supplemented with 10% of the SILS-IS preparation. The dilutions thus contained increasing amounts of the ^12^C-isotopomer nucleosides but constant amounts of ^13^C-isotopomer nucleosides, and were analyzed using the dMRM method adapted to side-by-side detection of ^12^C- and ^13^C-nucleosides. The nucleoside peaks were integrated for the external standard and the SIL-IS signal, and the ratio of the resulting areas used to calculate a separate NIF value for each dilution measurement. Figure 2B shows one exemplary set of results for Am. The upper left part of Figure 2B shows that the calibration curve obtained by plotting the peak area to the amount of injected, unlabeled Am has a sigmoid shape. The curve displays a linear dynamic range extending over four orders of magnitude, which is delimited in the LOD/LOQ range (Table 1) at the lower end, and by saturation of the MS at the upper end (1–10 pmol of analyte; gray in Figure 2B). This kind of calibration curve was obtained for all analyzed nucleosides.

Despite constant amounts of SIL-IS in all dilutions, decreased peak areas (gray areas in Figure 2B) were observed for ^13^C-derived signals to an identical extent as for ^12^C-derived signals at high analyte concentrations, presumably caused by ion suppression due to high concurrent amounts of unlabeled nucleoside. Since the effect equally affects both types of isotopomers, it is cancelled out in the NIF and a plot of NIF values shows a linear increase over six orders of magnitude, effectively extending the linear dynamic range in measurements by two orders of magnitude at the upper concentration range (see right graph of Figure 2B). Linear fit of this dataset shows a clear correlation of the labeled and unlabeled nucleosides, even in the range of saturation (*R*^2^ = 0.999). The slope is henceforth used as the relative response factor for nucleosides (rRFN) that relates the signal of a nucleosides to its injected amount using the signal of its ^13^C-isotopomer in the SIL-IS, as is illustrated in Equation (1). Respective rRFN values for all nucleosides are shown in Table 2. These values now allow calculation of the absolute amount of any of the modified nucleosides in Table 2 from a sample that has been supplemented with a defined amount of SIL-IS before analysis. Importantly, thus calculated values for absolute amounts are not affected by instrumental or by sample parameters anymore.

(1) \begin{eqnarray*} &&{{{\rm Nucleoside}\;{\rm of}\;{\rm interest}\,({\rm pmol})}} \nonumber\\ &&\!\!= \frac{{{\rm signal}({\rm nucleoside}\;{\rm of}\;{\rm interest})}}{{{\rm rRFN}({\rm {\rm nucleoside}}\;{\rm of}\;{\rm interest}) {\times} {\rm signal}({\rm SIL-IS})}} \end{eqnarray*}

**Table 2.** Relative response factors (rRFN)

Modified nucleoside	rRFN (pmol^−1^)	RSD^a^ (%)
Cytidines		
2′-O-Methylcytidine	0.00540	3.04
5-Methylcytidine	0.62794	2.36
Uridines		
Pseudouridine	0,00022	1.51
5-Methyluridine	0.00038	1.90
Guanosines		
7-Methylguanosine	0.00021	3.06
2′-O-methylguanosine	0.00179	1.53
Adenosines		
*N6*-Methyladenosine	0.01929	1.71
2′*-*O-methyladenosine	0.04538	1.69
*N6*-dimethyladenosine	0.89325	3.63
Inosine	0.00793	0.81

Factors are valid in the range of 1 fmol–100 pmol (exception m^6^A: 1 fmol–10 pmol).

^a^The RSD values were obtained over a 12-week period and are identical to those shown in Figure 4.

### UV-based quantification is necessary to determine the amount of injected RNA

One of the most sought after and exigent form of quantification is the determination of modification yields, e.g. the fraction of modified nucleoside in relation to the parent nucleoside. To this end, it is necessary to correlate the absolute amount of detected modified ribonucleoside to the absolute amount of injected sample. However, MS/MS quantification of the major nucleosides is problematic, since their frequency typically exceeds that of modified nucleosides by at least two orders of magnitude. Consequently, their MS signal typically reaches the saturation zone (compare gray area in Figure 2B) and thus leads to overestimation of the modification yield. UV detection at 254 nm offers a good solution to this problem, since saturation of the UV absorption occurs only at concentrations, which are at least three orders of magnitude higher. By performing calibration measurements for cytidine, uridine, guanosine and adenosine, UV response factors (UVF) were obtained, which essentially reflect the UV-extinction coefficient *ϵ*^254^, and which relate the UV signal to absolute amounts of major nucleosides. Care must be exercised when subtracting the UV signal contributed by the SIL-IS. Typical modification yields are reported in % modification of parent nucleoside (e.g. %m^5^C of C (21)) when mixtures of unknown sequence and composition are analyzed. However, for samples with a defined sequence, it is of interest to express the modification yield in mol% keeping an eye on the number of presumed modification sites and the degree of their occupancy. This is simply a normalization of the above percentage to the number of parent nucleosides in the known sequence. Interestingly, in the process of several such analyses, we noticed that normalization to the guanosine UV signal resulted in the most stable results (not shown). Although we have no explanation at this point, we suggest the guanosine-based calculation based on our experiences. Given a defined RNA sequence, the yield can be backtracked and expressed in mol% also for non-guanosine parent nucleosides. An example is given in the following section.

### Application to an *in vitro* assay for pseudouridine formation

**Figure 3.**
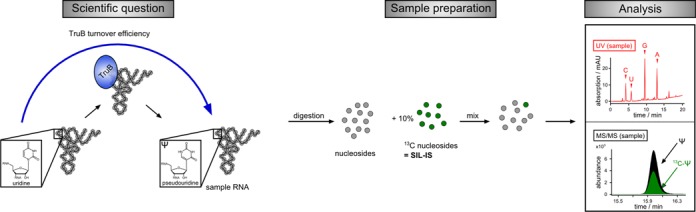
Determination of TruB turnover completeness. *Saccharomyces cerevisiae* tRNA^Phe^ transcript was incubated with TruB, digested and SIL-IS added to determine the absolute amount of pseudouridine formed. For analysis, the MS/MS traces were used to calculate the amount of injected pseudouridine in the sample. These results were compared to the amount of injected RNA, which is received by analysis of the UV chromatogram at 254 nm. Thereby, a turnover efficiency of 99.8% was found for TruB/*S.cerevisiae* tRNA^Phe^. Numeric details and a flow chart are given in Supplementary Table S2 and Supplementary Figure S1.

An application of the newly established SIL-IS approach is demonstrated in Figure 3. Our example reflects a typical *in vitro* assay, where an *S. cerevisiae* tRNA^Phe^ transcript was incubated with the pseudouridine synthase TruB, which is responsible for uridine to pseudouridine (Ψ) isomerization at position 55 of the tRNA (34). The sample was digested, supplemented with 10% of SIL-IS, and analyzed. The MS/MS traces of ^13^C Ψ and unlabeled Ψ were used to calculate the NIF. The NIF was then divided by the rRFN (from the calibration database) and revealed an absolute amount of ∼13 pmol Ψ in the sample. To assess the amount of RNA in the injected sample fraction, the UV chromatogram recorded at 254 nm was used. The peak for guanosine was integrated and the SIL-IS guanosine peak subtracted to give the UV area of the sample guanosine. The sample UV area was then correlated with the UV-response factor of guanosine, UVF(G), and the numbers of guanosines in the sample tRNA (23G). From these calculations, the amount of injected RNA was determined to be 13.2 pmol. A direct correlation of Ψ to RNA indicated a modification efficiency of 99.8%. The workflow including calculation steps is depicted in Figure 3 and Supplementary Figure S1. For independent verification, an aliquot of the sample was supplemented with 100 fmol Ψ. Analysis of the spiked sample revealed a turnover of 101% which is in the range of the 2% standard deviation of the Ψ − rRFN. The complete data processing of the spiked and unspiked sample using all major nucleosides yielded similar turnover efficiencies (for details see the Supplementary information).

### Comparison of relative and absolute quantification

**Figure 4.**
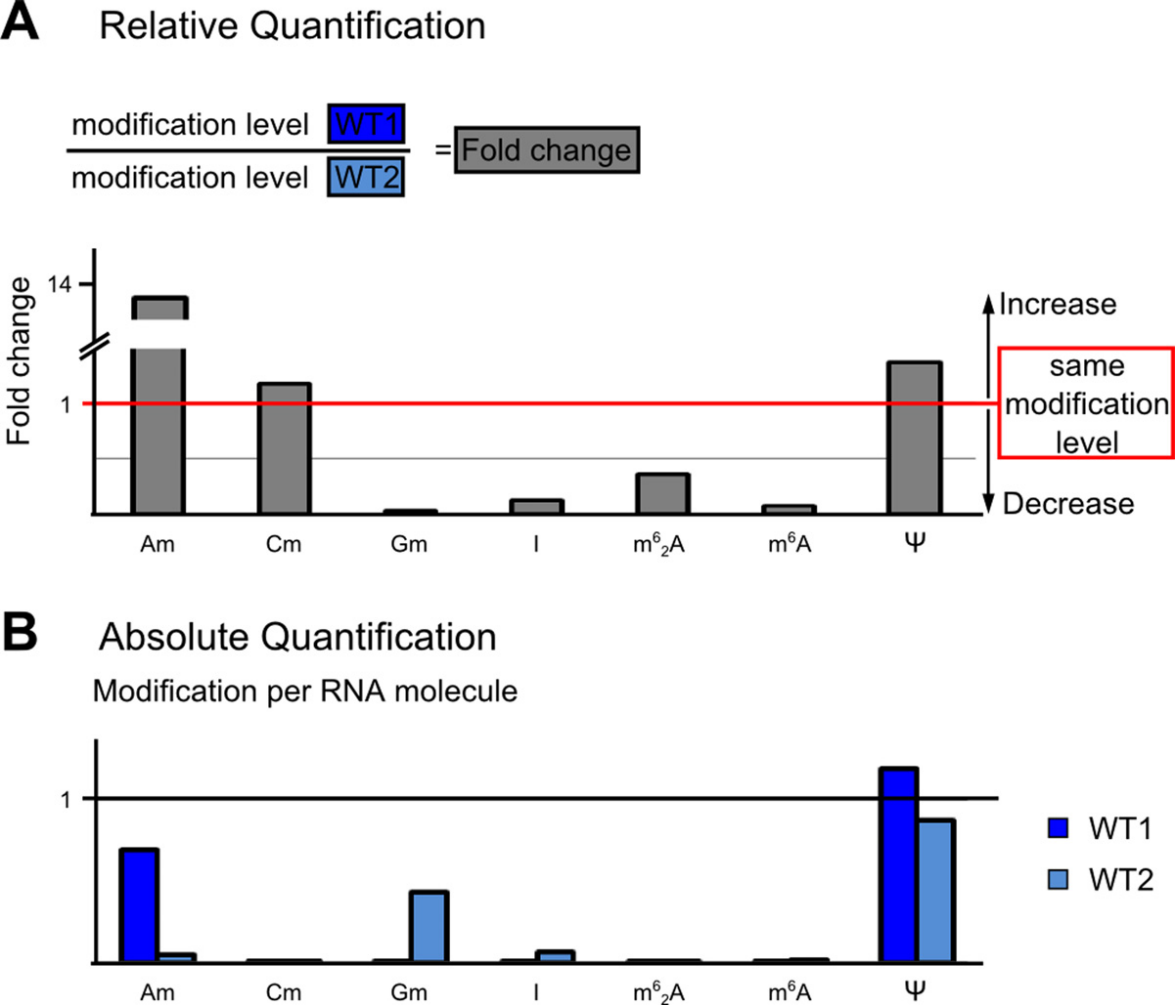
Relative and absolute quantifications of ribosomal RNA modifications. (**A**) Relative quantification displayed as fold changes for several modified nucleosides. The fold changes are the ratio of the modification level of rRNA fragments from WT1 and WT2. Fold changes <1 indicate higher levels in WT2 whereas fold changes >1 indicate higher modification levels in WT1. (**B**) Absolute modification of modified nucleosides from the same analysis using rRFN values. Am, Gm and Ψ are present in stoichiometrically relevant numbers whereas Cm, I, m^6^_2_A and m^6^A are only present in traces.

The example analysis above demonstrates the accuracy of our established internal standard for a single modified nucleoside. In an additional study, we have used our approach to compare the levels of several modifications of two ribosomal RNA fragments derived from two different *S. cerevisiae* strains (WT1 and WT2 (13)) both in a relative and in an absolute quantification. The relative quantification in Figure 4A shows significantly increased levels in Am and decreased levels for Gm, I, m^6^_2_A and m^6^A in WT1 samples compared to WT2, while pseudouridine and Cm seem to have similar modification levels. The absolute quantification results in Figure 4B reveals that the rRNA fragment does not contain I, m^6^_2_A and m^6^A in stoichiometrically relevant numbers and therefore the fold changes for I, m^6^_2_A and m^6^A are an artifact arising from background noise. However, for Am the increase in the fold-change value is clearly relevant (13) as the modification level for WT1 is nearly 68%. This comparative analysis also reveals higher Gm levels in the WT2 rRNA fragment, which makes it an interesting target for further analysis in future studies. The present results demonstrate the importance of absolute quantification as a tool to evaluate the numbers received by relative quantification and asses the significance of such fold changes.

### Reproducibility of relative response factors over 12 weeks

**Figure 5.**
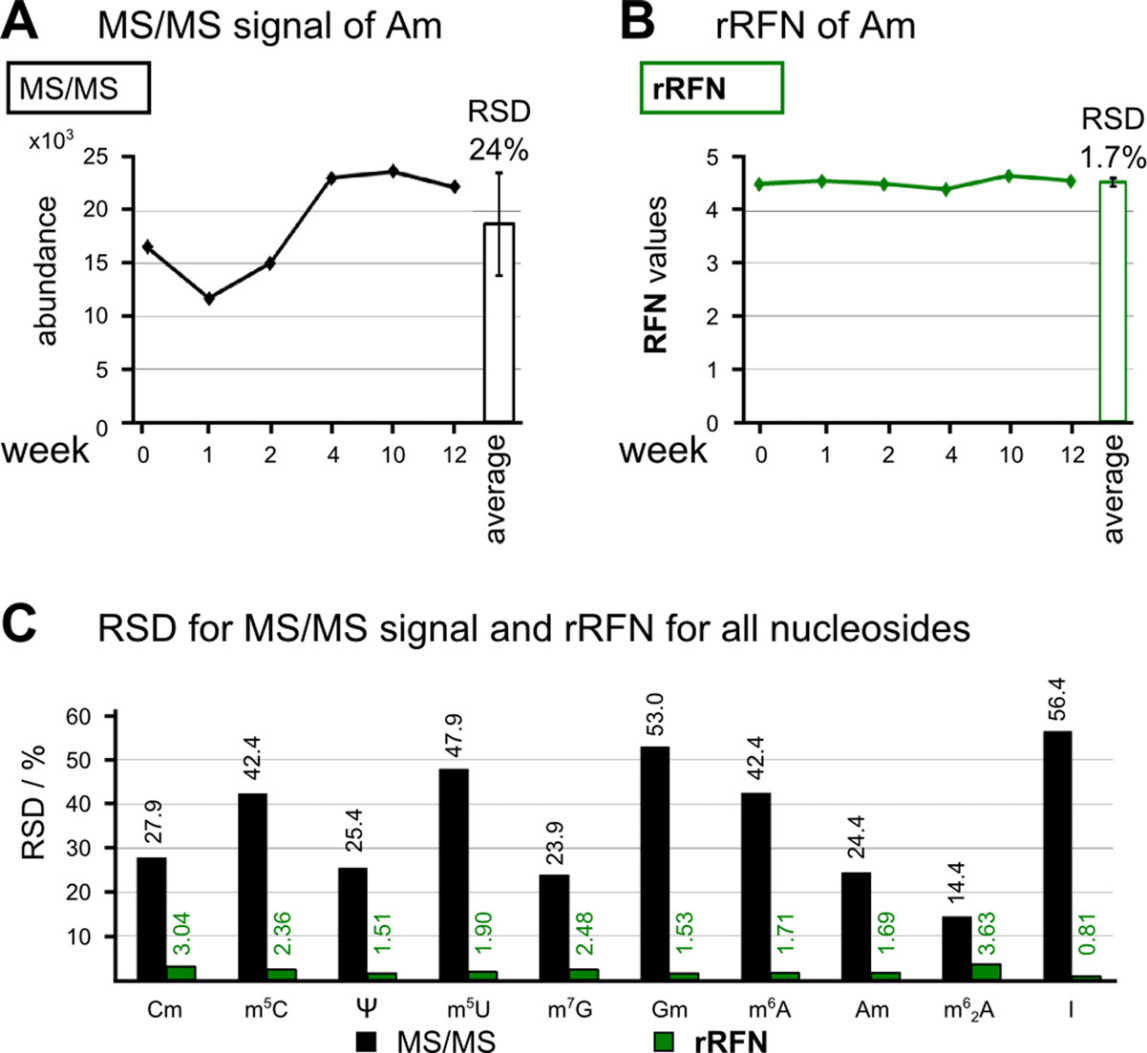
Reproducibility of rRFN values over 12 weeks. Relative standard deviations (RSDs) in % for the MS/MS signal and the isotope normalized signal of 2’-O-methyladenosine (Am) over a period of 12 weeks. (**A**) MS/MS abundance of constant amounts and average abundance with RSD. (**B**) Relative response factor (rRFN) of Am obtained by the measurements shown in (A). (**C**) RSD of MS/MS signal and rRFN for 10 modified ribonucleosides after 12 weeks. In black, the RSD in % of the MS abundance without ^13^C SIL-IS-based correction is shown. RSDs of the respective rRFN are in green.

To assess the capability of thus established rRFN values to equalize fluctuations of instrumental parameters occurring on a larger timeframe, we analyzed the aforementioned calibration solutions after 1, 2, 4, 10 and 12 consecutive weeks, during which the instrument was used for routine analysis of other nucleoside samples. rRFN values were established at these time points and compared to the uncorrected MS/MS signal. The variations of the latter, as plotted in Figure 5A for the example of Am, were subject to strong fluctuations of instrumental parameters which culminate in an RSD of 24%, while the corresponding fluctuations of the rRFN are <2%. Thus, as expected, detection of both the unlabeled and the corresponding isotopomer is strongly influenced by instrumental parameters, resulting in the MS signal fluctuations. These fluctuations are very efficiently equalized by the usage of the nucleoside–isotope ratio for all examined modifications. Figure 5B summarizes the RSD for all analyzed modified nucleosides. While the RSD ranges from a minimum of 14 to 56% for the MS signal fluctuation, RSD values for the rRFN are all <4%, and, with three exceptions (Cm, m^6^_2_A and m^7^G) even <2%. We conclude that establishing an rRFN allows quantification of samples by normalizing them to the SIL-IS, even for measurements effected at large time intervals.

## SUMMARY AND CONCLUSION

By feeding ^13^C-labeled glucose to *E. coli* and thus exploiting the parallel biosynthesis of a plethora of modified nucleosides, we have circumvented chemical synthesis of individual isotopomers and generated an SIL-IS in the very short turnaround time of a few days. In comparison to a ^15^N SIL-IS, which we have generated simultaneously (20), the ^13^C-isotopomers yielded better signal separation between unlabeled sample and SIL-IS because of the higher number of carbon atoms in nucleosides compared to nitrogen atoms. We have eschewed the use of deuterium-labeled compounds, since the long-term stability against proton exchange in aqueous solution is poorly understood. Of particular importance, the ^13^C-SIL-IS is suitable for relative quantification of two (or more) samples without further calibration, because it reliably reports the relative amounts of analyte and standard for each given modification in each sample. By normalizing signals to the SIL-IS, the variability related to sample, instrumental and physicochemical parameters were equalized, and therefore the critical parameter rRFN, once determined in a calibration run, is stable for several months.

An application to a typical *in vitro* biochemical assay was demonstrated. Such assays were typically performed by TLC analysis of a nucleotide digest of RNA transcripts prelabeled by *in vitro* transcription in the presence of α-^32^P-UTP (39). Such protocols are very demanding in terms of turnaround and hands-on time, and thus LC–MS-based quantification using our SIL-IS presents an attractive alternative of similar sensitivity. In comparison to spike-in calibration, our method presents several significant advantages, in particular that the calibration measurements can be performed independently of the actual measurements. Also, our method consumes a lot less material, since spike-in calibration requires sample for every calibration point, unless one-point calibrations are used, which in turn sacrifices accuracy. A further application example highlights the advantages of performing an absolute quantification in addition to solely determining the relative modification of two samples. Here, the absolute quantification identified artifacts resulting from forming the ration of two values near the background level. However, elimination of such artifacts should also be feasible without absolute quantification, simply by determining standard deviations of multiple biological replicates.

By application of the SIL-IS, a remarkable reduction of the RSD to <4%, measured over a period of 3 months was achieved. Determination of absolute amounts of modifications can be performed with LOQs in the single-digit femtomole range. It requires a more sophisticated combination of external calibration, validation and spike-in methods, and a somewhat complex series of calculations to eliminate the detrimental influence of a maximum number of parameters. Obviously, with each new batch, preparation of SIL-IS calibration has to be repeated, and it is therefore meaningful to prepare it on a large scale. In this respect, it is important to keep in mind that certain modifications appear to be unstable when unfrozen (data not shown). Of the parameters that may negatively affect quantification, sample contaminations with unknown substances that cause signal depression are currently the most pressing problem. Indeed, we have encountered many analytical problems in the recent past, where such contaminations made accurate assessment of the modification content critically dependent on the application of this SIL-IS.

## SUPPLEMENTARY DATA

Supplementary Data are available at NAR Online.

SUPPLEMENTARY DATA

## References

[1] El Yacoubi B., Bailly M., de Crecy-Lagard V. (2012). Biosynthesis and function of posttranscriptional modifications of transfer RNAs. Annu. Rev. Genet..

[2] Motorin Y., Helm M. (2011). RNA nucleotide methylation. Wiley Interdiscip. Rev. RNA.

[3] Meyer K.D., Saletore Y., Elemento O., Mason C.E., Jaffrey S.R. (2012). Comprehensive analysis of mRNA methylation reveals enrichment in 3′ UTRs and near stop codons. Cell.

[4] Dominissini D., Moshitch-Moshkovitz S., Schwartz S., Salmon-Divon M., Ungar L., Osenberg S., Cesarkas K., Jacob-Hirsch J., Amariglio N., Kupiec M. (2012). Topology of the human and mouse m6A RNA methylomes revealed by m6A-seq. Nature.

[5] Karijolich J., Yu Y.T. (2011). Converting nonsense codons into sense codons by targeted pseudouridylation. Nature.

[6] Khoddami V., Cairns B.R. (2013). Identification of direct targets and modified bases of RNA cytosine methyltransferases. Nat. Biotechnol..

[7] Amort T., Soulière MF., Wille A., Jia X.Y., Fiegl H., Wörle H., Micura R., Lusser A. (2013). Long non-coding RNAs as targets for cytosine methylation. RNA Biol..

[8] Chan C.T., Dyavaiah M., DeMott M.S., Taghizadeh K., Dedon P.C., Begley T.J. (2010). A quantitative systems approach reveals dynamic control of tRNA modifications during cellular stress. PLoS Genet..

[9] Jia G., Fu Y., Zhao X., Dai Q., Zheng G., Yang Y., Yi C., Lindahl T., Pan T., Yang Y.G. (2011). N6-methyladenosine in nuclear RNA is a major substrate of the obesity-associated FTO. Nat. Chem. Biol..

[10] Zheng G., Dahl J.A., Niu Y., Fedorcsak P., Huang C.M., Li C.J., Vågbø C.B., Shi Y., Wang W.L., Song S.H. (2013). ALKBH5 is a mammalian RNA demethylase that impacts RNA metabolism and mouse fertility. Mol. Cell.

[11] Brulé H., Holmes W.M., Keith G., Giegé R., Florentz C. (1998). Effect of a mutation in the anticodon of human mitochondrial tRNAPro on its post-transcriptional modification pattern. Nucleic Acids Res..

[12] Helm M., Florentz C., Chomyn A., Attardi G (1999). Search for differences in post-transcriptional modification patterns of mitochondrial DNA-encoded wild-type and mutant human tRNALys and tRNALeu(UUR). Nucleic Acids Res..

[13] Buchhaupt M., Sharma S., Kellner S., Oswald S., Paetzold M., Peifer C., Watzinger P., Schrader J., Helm M., Entian KD. (2014). Partial methylation at Am100 in 18S rRNA of baker's yeast reveals ribosome heterogeneity on the level of eukaryotic rRNA modification. PLoS One.

[14] Addepalli B., Limbach P.A. (2011). Mass spectrometry-based quantification of pseudouridine in RNA. J. Am. Soc. Mass Spectrom..

[15] Li S., Limbach P.A. (2013). Mass spectrometry sequencing of transfer ribonucleic acids by the comparative analysis of RNA digests (CARD) approach. Analyst.

[16] Popova A.M., Williamson J.R. (2014). Quantitative analysis of rRNA modifications using stable isotope labeling and mass spectrometry. J. Am. Chem. Soc..

[17] Zhang J.J., Zhang L., Zhou K., Ye X., Liu C., Zhang L., Kang J., Cai C. (2011). Analysis of global DNA methylation by hydrophilic interaction ultra high-pressure liquid chromatography tandem mass spectrometry. Anal. Biochem..

[18] Nikcevic I., Wyrzykiewicz T.K., Limbach P.A. (2011). Detecting low-level synthesis impurities in modified phosphorothioate oligonucleotides using liquid chromatography–high resolution mass spectrometry. Int. J. Mass Spectrom..

[19] Su D., Chan C.T., Gu C., Lim K.S., Chionh Y.H., McBee M.E., Russell B.S., Babu I.R., Begley T.J., Dedon P.C. (2014). Quantitative analysis of ribonucleoside modifications in tRNA by HPLC-coupled mass spectrometry. Nat. Protoc..

[20] Kellner S., Neumann J., Rosenkranz D., Lebedeva S., Ketting R.F., Zischler H., Schneider D., Helm M. (2014). Profiling of RNA modifications by multiplexed stable isotope labelling. Chem. Commun. (Camb.).

[21] Tuorto  F., Liebers R., Musch T., Schaefer M., Hofmann S., Kellner S., Frye M., Helm M., Stoecklin G., Lyko F. (2012). RNA cytosine methylation by Dnmt2 and NSun2 promotes tRNA stability and protein synthesis. Nat. Struct. Mol. Biol..

[22] Brandmayr C., Wagner M., Brückl T., Globisch D., Pearson D., Kneuttinger A.C., Reiter V., Hienzsch A., Koch S., Thoma I. (2012). Isotope-based analysis of modified tRNA nucleosides correlates modification density with translational efficiency. Angew. Chem. Int. Ed. Engl..

[23] Chen M.L., Shen F., Huang W., Qi J.H., Wang Y., Feng Y.Q., Liu S.M., Yuan B.F. (2013). Quantification of 5-methylcytosine and 5-hydroxymethylcytosine in genomic DNA from hepatocellular carcinoma tissues by capillary hydrophilic-interaction liquid chromatography/quadrupole TOF mass spectrometry. Clin. Chem..

[24] Contreras-Sanz A., Scott-Ward T.S., Gill H.S., Jacoby J.C., Birch R.E., Malone-Lee J., Taylor K.M., Peppiatt-Wildman C.M., Wildman S.S. (2012). Simultaneous quantification of 12 different nucleotides and nucleosides released from renal epithelium and in human urine samples using ion-pair reversed-phase HPLC. Purinergic Signal.

[25] Yan M., Wang Y., Hu Y., Feng Y., Dai C., Wu J., Wu D., Zhang F., Zhai Q. (2013). A high-throughput quantitative approach reveals more small RNA modifications in mouse liver and their correlation with diabetes. Anal. Chem..

[26] Dalluge J.J., Hashizume T., McCloskey J.A. (1996). Quantitative measurement of dihydrouridine in RNA using isotope dilution liquid chromatography-mass spectrometry (LC/MS). Nucleic Acids Res..

[27] Brückl T., Globisch D., Wagner M., Müller M., Carell T. (2009). Parallel isotope-based quantification of modified tRNA nucleosides. Angew. Chem. Int. Ed. Engl..

[28] Li J., Leung E.M., Choi M.M., Chan W. (2013). Combination of pentafluorophenylhydrazine derivatization and isotope dilution LC-MS/MS techniques for the quantification of apurinic/apyrimidinic sites in cellular DNA. Anal. Bioanal. Chem..

[29] Chang Y.L., Hsieh C.L., Huang Y.M., Chiou W.L., Kuo Y.H., Tseng M.H. (2013). Modified method for determination of sulfur metabolites in plant tissues by stable isotope dilution-based liquid chromatography-electrospray ionization-tandem mass spectrometry. Anal. Biochem..

[30] Hienzsch A., Deiml C., Reiter V., Carell T. (2013). Total synthesis of the hypermodified RNA bases wybutosine and hydroxywybutosine and their quantification together with other modified RNA bases in plant materials. Chemistry.

[31] Pearson D., Hienzsch A., Wagner M., Globisch D., Reiter V., Özden D., Carell T. (2011). LC-MS based quantification of 2′-ribosylated nucleosides Ar(p) and Gr(p) in tRNA. Chem. Commun. (Camb.).

[32] Cantara W.A., Crain P.F., Rozenski J., McCloskey J.A., Harris K.A., Zhang X., Vendeix F.A., Fabris D., Agris P.F. (2011). The RNA Modification Database, RNAMDB: 2011 update. Nucleic Acids Res..

[33] Hayrapetyan A., Grosjean H., Helm M. (2009). Effect of a quaternary pentamine on RNA stabilization and enzymatic methylation. Biol. Chem..

[34] Pan H., Agarwalla S., Moustakas D.T., Finer-Moore J., Stroud R.M. (2003). Structure of tRNA pseudouridine synthase TruB and its RNA complex: RNA recognition through a combination of rigid docking and induced fit. Proc. Natl. Acad. Sci. U.S.A..

[35] Sharma S., Yang J., Watzinger P., Kötter P., Entian K.D. (2013). Yeast Nop2 and Rcm1 methylate C2870 and C2278 of the 25S rRNA, respectively. Nucleic Acids Res..

[36] Dayie T.K., Thakur C.S. (2010). Site-specific labeling of nucleotides for making RNA for high resolution NMR studies using an E. coli strain disabled in the oxidative pentose phosphate pathway. J. Biomol. NMR.

[37] Lu K., Miyazaki Y., Summers M.F. (2010). Isotope labeling strategies for NMR studies of RNA. J. Biomol. NMR.

[38] Duss O., Lukavsky P.J., Allain F.H. (2012). Isotope labeling and segmental labeling of larger RNAs for NMR structural studies. Adv. Exp. Med. Biol..

[39] Grosjean H., Droogmans L., Roovers M., Keith G. (2007). Detection of enzymatic activity of transfer RNA modification enzymes using radiolabeled tRNA substrates. Methods Enzymol..

