# Supplementary material for: Absolute and relative quantification of RNA modifications via biosynthetic isotopomers
Source: Nucleic Acids Res. 2014 Aug 16;42(18):e142. doi: 10.1093/nar/gku733 (PMC4191383; doi:10.1093/nar/gku733)
Supplement: SUPPLEMENTARY DATA [file supp_42_18_e142__index.html]

Absolute and relative quantification of RNA modifications via biosynthetic isotopomers — Absolute and relative quantification of RNA modifications via biosynthetic isotopomers — SUPPLEMENTARY DATA 

# Absolute and relative quantification of RNA modifications *via* biosynthetic isotopomers

## SUPPLEMENTARY DATA

**Files in this Data Supplement:**

- SUPPLEMENTARY DATA
