# Supplementary material for: Absolute and relative quantification of RNA modifications via biosynthetic isotopomers
Source: Nucleic Acids Res. 2014 Aug 16;42(18):e142. doi: 10.1093/nar/gku733 (PMC4191383; doi:10.1093/nar/gku733)

Absolute and relative quantification of RNA modifications *via* biosynthetic isotopomers

Stefanie Kellner^1^, Antonia Ochel^1^, Kathrin Thüring^1^, Felix Spenkuch^1^,
Jennifer Neumann^1^, Sunny Sharma^2^, Karl-Dieter Entian^2^, Dirk Schneider^1^ & Mark Helm^1^*

^1^Institute of Pharmacy and Biochemistry, Johannes Gutenberg University Mainz, Mainz, Germany, ^2^Institute for Molecular Biosciences, Johann-Wolfgang Goethe University, Frankfurt am Main, Germany

* Phone: +49-6131-3925731. Fax: +49-6131-3920373. Email: mhelm@uni-mainz.de

**Supplementary information**

**Table S1:** Parameters for dynamic MRM method

**Table S2:** Quantification of pseudouridine for determination of TruB turnover

**Figure S1:** Flow chart for determination of TruB turnover completeness

**Table S1** QQQ parameters of dynamic MRM method (green: isotopologues of natural nucleosides)

| Compound | Precursor Ion | Product Ion | Fragmentor | Collision Energy | Cell Accelerator Voltage | Retention time [min] | Retention time window [min] |
| --- | --- | --- | --- | --- | --- | --- | --- |
| A | 268 | 136 | 80 | 10 | 2 | 13.3 | 3 |
| Am | 282 | 136 | 92 | 13 | 2 | 15.9 | 1 |
| Am ^13^C | 293 | 141 | 92 | 13 | 2 | 15.9 | 1 |
| C | 244 | 112 | 60 | 10 | 2 | 4.7 | 3 |
| Cm | 258 | 112.1 | 60 | 9 | 2 | 9.2 | 1 |
| Cm ^13^C | 268 | 116.1 | 60 | 9 | 2 | 9.2 | 1 |
| D ^13^C | 256 | 119 | 80 | 10 | 2 | 3.5 | 1 |
| G | 284 | 152 | 80 | 10 | 2 | 10.0 | 3 |
| Gm | 298 | 152 | 72 | 5 | 2 | 12.3 | 1 |
| Gm ^13^C | 309 | 157 | 72 | 5 | 2 | 12.3 | 1 |
| hm^5^C | 274.2 | 142.1 | 80 | 10 | 2 | 5.2 | 5 |
| I | 269 | 137 | 76 | 5 | 2 | 9.5 | 1 |
| I ^13^C | 279 | 142 | 76 | 5 | 2 | 9.5 | 1 |
| m^1^A | 282 | 150 | 92 | 17 | 2 | 7.2 | 1 |
| m^1^A ^13^C | 293 | 156 | 92 | 17 | 2 | 7.2 | 1 |
| m^1^G ^13^C | 309 | 172 | 82 | 9 | 2 | 12.3 | 1 |
| m^6^_2_A | 296 | 164.1 | 102 | 17 | 2 | 18.6 | 1 |
| m^6^_2_A ^13^C | 308 | 171 | 102 | 17 | 2 | 18.6 | 1 |
| m^2^A ^13^C | 293 | 156 | 92 | 17 | 2 | 16.5 | 1 |
| m^2^G | 298 | 166.1 | 82 | 9 | 2 | 12.6 | 1 |
| m^2^G ^13^C | 309 | 172 | 82 | 9 | 2 | 12.6 | 1 |
| m^3^C ^13^C | 268 | 131 | 40 | 9 | 2 | 5.4 | 5 |
| m^5^C | 258 | 126.1 | 40 | 9 | 2 | 8.4 | 1 |
| m^5^C ^13^C | 268 | 131 | 40 | 9 | 2 | 8.4 | 1 |
| m^5^s^2^U | 275 | 143 | 66 | 5 | 2 | 13.6 | 1 |
| m^5^s^2^U ^13^C | 285 | 148 | 66 | 5 | 2 | 13.6 | 1 |
| m^5^U | 259 | 127 | 76 | 5 | 2 | 10.0 | 1 |
| m^5^U ^13^C | 269 | 132 | 76 | 5 | 2 | 10.0 | 1 |
| m^5^UD_4_ | 263.2 | 131.1 | 76 | 5 | 2 | 10.4 | 1 |
| m^6^A | 282 | 150.1 | 92 | 17 | 2 | 16.6 | 1 |
| m^6^A ^13^C | 293 | 156 | 92 | 17 | 2 | 16.6 | 1 |
| m^7^G | 298 | 166.1 | 82 | 9 | 2 | 8.9 | 1 |
| m^7^G ^13^C | 309 | 172 | 82 | 9 | 2 | 8.9 | 1 |
| Ψ | 245 | 209 | 81 | 5 | 2 | 3.7 | 1 |
| Ψ ^13^C | 254 | 218 | 81 | 5 | 2 | 3.7 | 1 |
| s^2^C ^13^C | 269 | 132 | 40 | 10 | 2 | 8.6 | 5 |
| s^2^U | 261 | 129 | 66 | 5 | 2 | 10.6 | 5 |
| s^4^U | 261 | 129 | 66 | 5 | 2 | 11.5 | 5 |
| s^4^U ^13^C | 270 | 133 | 66 | 5 | 2 | 11.5 | 5 |
| U | 245 | 113 | 92 | 13 | 2 | 6.3 | 3 |
| Um | 259 | 113 | 66 | 5 | 2 | 10.9 | 1 |
| Um ^13^C | 269 | 117 | 66 | 5 | 2 | 10.9 | 1 |

**Quantification of TruB turnover efficiency**

RNA composition: 18C, 17(16U, 1Ψ) U, 23G and 18A

UV-Faktoren: C/1.117, U/1.5921, G/2.2002 and A/2.2102

rRFN (Ψ) = 0.000223

**Table S2:** Quantification of TruB turnover in unspiked and spiked samples

| Raw data MS | |  |  |  |  |
| --- | --- | --- | --- | --- | --- |
|  | Name | m/z (precursor) | m/z (product) | RT | Area |
| Psi spike | Pseudouridine C13 | 254 | 218 | 3.627 | 24442 |
|  | Pseudouridine | 245 | 209 | 3.619 | 524 |
|  |  |  |  |  |  |
| TruB no spike | Pseudouridine C13 | 254 | 218 | 3.612 | 5165 |
|  | Pseudouridine | 245 | 209 | 3.616 | 4290 |
|  |  |  |  |  |  |
| TruB spike | Pseudouridine C13 | 254 | 218 | 3.609 | 5280 |
|  | Pseudouridine | 245 | 209 | 3.61 | 4347 |
|  |  |  |  |  |  |
| 1) Raw data UV and RNA quantification | | |  |  |  |
| **UV areas** | **C** | **U** | **G** | **A** |  |
| Psi spike | 72.59 | 48.97 | 134 | 105 |  |
|  |  |  |  |  |  |
|  |  |  |  |  |  |
| TruB no spike | 145 | 135 | 323 | 258 |  |
| without ISTD | 72.41 | 86.03 | 189 | 153 |  |
| Main nuc [pmol] | 64.82 | 54.03 | 85.90 | 69.22 |  |
| TruB spike | 140.5 | 130 | 314 | 250 |  |
| without ISTD | 67.91 | 81.03 | 180 | 145 |  |
| Main nuc [pmol] | 60.79 | 50.89 | 81.81 | 65.60 |  |
| 2) Quantification Modification | |  |  |  |  |
|  | **NIF** | **NIF/rRFN** |  | quota |  |
| Psi spike | 0.0214 | 96.1368 | fmol | 100.0000 | fmol |
|  |  |  |  |  |  |
| TruB no spike | 0.8306 | 3724.62 | fmol | 3724.6211 | fmol |
|  |  |  |  |  |  |
| TruB spike | 0.8233 | 3691.90 | fmol | 3591.9079 | fmol |
| 3) Turnover efficiency in % (calculated for each major nucleoside) | | | | | |
|  | **for G** | **for A** | **for C** | **for U** |  |
| TruB no spike | 99.72 | 96.84 | 103.42 | 110.29 |  |
|  |  |  |  |  |  |
| TruB spike | 100.98 | 98.55 | 100.43 | 112.91 |  |

**Figure S1:** Flow chart for determination of TruB turnover completeness


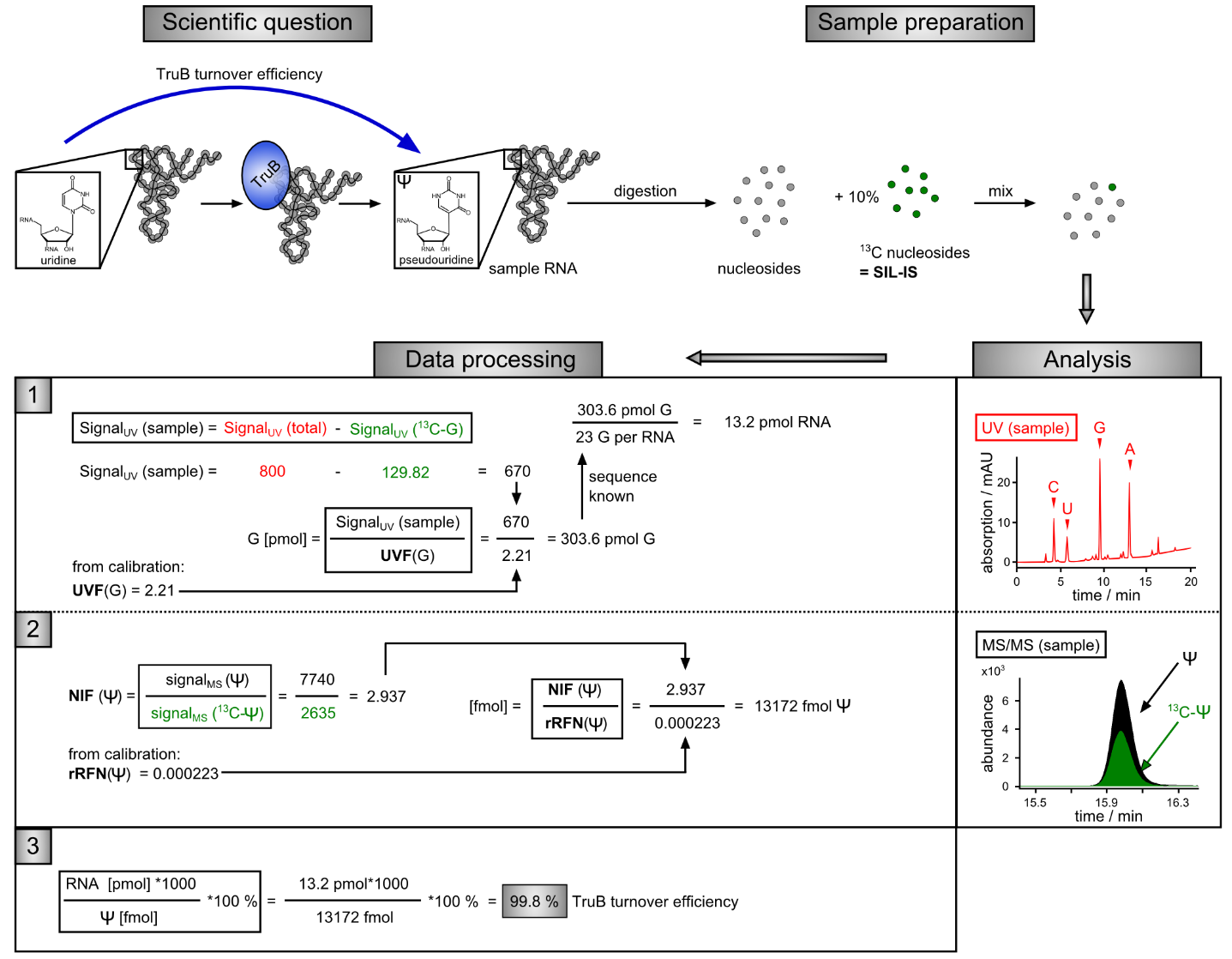

Supplement: SUPPLEMENTARY DATA [file supp_gku733_nar-01336-met-g-2014-File007.docx]
